# miR-29b, miR-205 and miR-221 Enhance Chemosensitivity to Gemcitabine in HuH28 Human Cholangiocarcinoma Cells

**DOI:** 10.1371/journal.pone.0077623

**Published:** 2013-10-17

**Authors:** Kinya Okamoto, Kenichi Miyoshi, Yoshikazu Murawaki

**Affiliations:** Second Department of Internal Medicine, Tottori University School of Medicine, Yonago, Tottori, Japan; Virginia Commonwealth University, United States of America

## Abstract

**Background and Aims:**

Cholangiocarcinoma (CCA) is highly resistant to chemotherapy, including gemcitabine (Gem) treatment. MicroRNAs (miRNAs) are endogenous, non-coding, short RNAs that can regulate multiple genes expression. Some miRNAs play important roles in the chemosensitivity of tumors. Here, we examined the relationship between miRNA expression and the sensitivity of CCA cells to Gem.

**Methods:**

Microarray analysis was used to determine the miRNA expression profiles of two CCA cell lines, HuH28 and HuCCT1. To determine the effect of candidate miRNAs on Gem sensitivity, expression of each candidate miRNA was modified via either transfection of a miRNA mimic or transfection of an anti-oligonucleotide. Ontology-based programs were used to identify potential target genes of candidate miRNAs that were confirmed to affect the Gem sensitivity of CCA cells.

**Results:**

HuCCT1 cells were more sensitive to Gem than were HuH28 cells, and 18 miRNAs were differentially expressed whose ratios over ± 2log2 between HuH28 and HuCCT1. Among these 18 miRNAs, ectopic overexpression of each of three downregulated miRNAs in HuH28 (miR-29b, miR-205, miR-221) restored Gem sensitivity to HuH28. Suppression of one upregulated miRNA in HuH28, miR-125a-5p, inhibited HuH28 cell proliferation independently to Gem treatment. Selective siRNA-mediated downregulation of either of two software-predicted targets, PIK3R1 (target of miR-29b and miR-221) or MMP-2 (target of miR-29b), also conferred Gem sensitivity to HuH28.

**Conclusions:**

miRNA expression profiling was used to identify key miRNAs that regulate Gem sensitivity in CCA cells, and software that predicts miRNA targets was used to identify promising target genes for anti-tumor therapies.

## Introduction

Cholangiocarcinoma (CCA) is a malignant cancer originating from the neoplastic transformation of biliary epithelial cells, and the incidence and prevalence of CCA are increasing progressively [1]. CCA is most often diagnosed at an advanced stage with intrahepatic and lymph-node metastases because the early stages of CCA progression are largely asymptomatic and effective screening biomarkers have not been developed [2]. To make matters worse, CCA is very resistant to common chemotherapies; the prognosis associated with unresectable CCA remains poor, and median overall survival is less than 12 months in these CCA cases [2], [3].

Gemcitabine (Gem; 2′,2′-difluorodeoxycytidine, dFdC) is a self-potentiating cytidine analogue and widely used as an anticancer agent [4]. Gem is transported into cells by concentrative nucleoside transporter 1 (gene symbol: SLC28A1) and equilibrative nucleoside transporter 1 (gene symbol: SLC29A1) mainly. Intracellular deoxycytidine kinase (gene symbol: DCK) metabolizes Gem to the active diphosphate (dFdC-DP) and triphosphate (dFdC-TP) nucleosides. dFdC-DP inhibits ribonucleotide reductase (RNR; constructed from RNR1 (gene symbol: RRM1) and RNR2 subunits (gene symbol: RRM2)), resulting in decreasing the concentration of deoxynucleoside triphosphates including deoxycytidine triphosphate (dCTP). dFdC-TP competes with dCTP for incorporation into DNA. The reduced intracellular concentration of dCTP potentiates the incorporation of dFdC-TP into DNA. After dFdC-TP incorporation, one more nucleotide is incorporated and DNA synthesis is completely inhibited. This process blocks the progression of cells through the G1/S-phase and eventually resulting in apoptosis. Export of Gem or its phosphorylated metabolites to the extracellular space is mainly mediated by multidrug resistance protein 5 (gene symbol: ABCC5). Gem-based treatments, either as monotherapy or in combination with other agents, have been suggested as alternative treatments for patients with unresectable CCA [3]. Hence the increasing the sensitivity of CCA to Gem treatment is urgently needed.

Micro-RNAs (miRNAs) are a class of endogenous, noncoding, small RNAs of 19 – 25 nucleotides (nt) that regulate gene expression [5]. Mature miRNAs are cleaved from 70- to 100- nt hairpin pre-microRNA precursors and are introduced into RNA induced silencing complexes (RISCs) [6]. A RISC bearing a miRNA usually binds to partially complementary sequence within the 3′ UTR region of a mRNA and thereby either represses the translation or induces the degradation of that mRNA. Because base-pairing over just 7 or 8 bases on miRNA seed region can elicit the effect of an miRNA, a single miRNA can regulate many target mRNAs [7], [8]. Owing to these features, miRNAs play an important role in many cellular processes, including those that are particularly important during carcinogenesis and tumor progression such as differentiation, proliferation, apoptosis, and stress responses [9]. Furthermore, more than 50% of the annotated human miRNA genes are located in regions that are amplified, deleted, translocated, or broken as fragile sites during the course of tumor development [10]. Accumulating evidence indicates that miRNAs are aberrantly expressed in myriad cancers and that these miRNA can function as oncogenes or tumor suppressors that affect carcinogenesis, tumor progression, and prognosis [11]. Some reports that focus on the sensitivity of cancer cells to chemotherapy agents indicate that expression levels of some miRNAs are related to the chemoresistance of malignant cells and that modification of the expression of these miRNA can restore chemosensitivity to these cancer cells [12], [13]. In this study, we examined the relationship between miRNA expression profile and sensitivity to Gem in two human CCA cell lines. In addition, we used ontology-filtering software to identify the gene targets of the miRNAs that had a role in conferring Gem sensitivity to CCA cells.

## Materials and Methods

### Cell lines and cultures

Both human intrahepatic CCA cell lines, HuCCT1 and HuH28, were purchased from Japan Health Science Research Resources Bank (Osaka, Japan). Each cell line was cultured in RPMI-1640 medium (Invitrogen, Life Technologies Corp., CA, USA) that contained 10% fetal bovine serum (Nichirei Bioscience, Tokyo, Japan) and in humidified conditions at 37 °C and 5% CO_2_. Antibiotics were not added to the culture medium when cells were prepared for transfection with miRNA mimics or oligonucleotides.

### Gem treatment

Gem hydrochloride was purchased from Wako (Osaka, Japan). A stock solution was prepared at 1 mmol/L (1×10^−3^ M) and was further diluted to anyone of several different final working concentrations from 1×10^−4^ to 1×10^−7^ M with cell culture medium that lacked antibiotics. Transfection of miRNA mimics, antisense oligonucleotides, or siRNA for miRNA target genes were performed 24 hr before the Gem treatment. All assays were conducted 72 hr after Gem treatment.

### Cell viability analysis

Cells were plated in 96-well plates at a density of 5.0×10^3^ cells per well. Cell viability was assessed 72 h after Gem treatment; the Cell Counting Kit-8 (CCK-8) (Dojindo, Kumamoto, Japan) and the manufacturer's protocol were used to assess cell viability.

### MicroRNA microarray analysis

The miRCURY™ LNA miRNA Arrays (Exiqon Inc., Vedbaek, Denmark) were used to determine the miRNA expression profile of each cell line. LNAs are a class of conformationally restricted nucleotide analogs that can increase the affinity of an oligonucleotide for its complementary miRNA. In brief, after the Agilent 2100 bio analyzer (Exiqon) was used to assess the quality of the total RNA preparations, 1-µg samples of total RNA were labeled using the Power Labeling kit (Exiqon). Hybridizations were performed on the miRCURY™ LNA miRNA Arrays ver. 11.0. For each sample, three independent hybridizations were performed on chips. The GenePix4000B® (Molecular Devices, CA, USA) was used to scan each microarray chip and to determine the signal intensities. The microarray data analysis tool ver. 3.2 (Filgen Inc., Aichi, Japan) was used to normalize and analyze miRNA expression levels.

### Microarray data deposition

The miRNA profiling by microarray results of HuCCT1 cells and HuH28 cells have been deposited in the NCBI Gene Expression Omnibus (http://www.ncbi.nlm.nih.gov/geo/) and are available under accession number GSE47396.

### Modification of miRNA expression and siRNA transfection

miRNA mimics, antisense oligonucleotides targeting miRNAs, and short interference RNAs (siRNAs) targeting the miRNA-target genes were purchased from Sigma-Aldrich (MO, USA), Ambion (TX, USA), or Invitrogen. Transfections were performed when cultures had reached 70% confluency in 96 well plates or 75 cm^2^ dishes; Lipofectamine RNAiMAX (Invitrogen) was used according to the manufacturer's instructions for all transfections. The final concentration of each miRNA mimic or siRNAs was 10 nM and that of each antisense oligonucleotide was 40 nM. Each experiment included three groups of control cells—untreated controls, mock-treated controls (receiving only transfection reagent), and a control treated with a non-silencing miRNA mimic or a negative control oligonucleotide. Stealth™ RNAi Negative Control (Invitrogen) and Anti-miR™ miRNA Inhibitors Negative Control #1 (Ambion) were used as the non-silencing control miRNA mimic and as the negative control antisense oligonucleotide, respectively.

### Predicting the target genes of miRNAs

Two different web-driven software programs, TargetScanHuman 5.1 (http://www.targetscan.org/) and DIANA-microT 4.0 (http://diana.cslab.ece.ntua.gr/) were used to predict the potential target genes of miRNAs that were identified as candidates in the search for miRNAs that affected the Gem resistance or sensitivity of CCA cells. Genes that were among the ontology-filtered results from TargetScanHuman and those from microT were designated putative target genes of candidate miRNAs. Ontology filtering was performed with web-driven gene ontology software DIANA mirPath (http://diana.cslab.ece.ntua.gr/). The following keywords—cancer, cell cycle, apoptosis, survival, cell signal pathways, pharmacokinetics, and drug metabolism— were used to select the chemosensitivity-related targets genes. The Gem metabolizing genes; SLC28A1, SLC29A1, DCK, RRM1, RRM2 and ABCC5 were also searched.

### Western-blot analysis

Total protein preparation and sodium lauryl sulfate - polyacrylamide gel electrophoresis (SDS-PAGE) were performed as described previously [14]. The separated proteins were transferred to polyvinylidene fluoride membranes using an iBlot ® (Invitrogen) gel transfer system. Membrane blocking and antibody binding were performed in a vacuum-driven incubator, SNAP i.d. ® (Merck Millipore, MA, USA). After being incubated in a pH 8.0 blocking buffer that contained 10 mM Tris, 150 mM NaCl, and 0.1% Tween 20 (TBST) and 0.1% low-fat powdered milk, membranes were incubated for 10 min with one or more primary antibodies against the following cellular proteins: c-KIT 1: 333 (Cell signaling technology Japan (CSTJ), Tokyo, Japan), dual specificity phosphatase 6 (DUSP6) 1: 167 (abcam), erythroblastic leukemia viral oncogene homolog 3 (ErbB3) 1: 333 (CSTJ) Leukemia inhibitory factor (LIF) 1: 200 (Santa Cruz Biotechnology CA, USA), Matrix metalloproteinase 2 (MMP-2) 1: 333 (CSTJ), Phosphoinositide-3-kinase regulatory subunit 1 (PIK3R1) 1: 333 (CSTJ), Vascular endothelial growth factor A (VEGFA) 1: 333 (abcam). For each experiment, β-actin 1: 833 (abcam) was used to as a loading control. Membranes were washed three times in TBST buffer, and then incubated with an appropriate secondary antibody conjugated to horseradish peroxidase (Sigma-Aldrich) for 10 min. The chemiluminescence reagent ECL prime (GE Healthcare Japan, Tokyo, Japan) was used to label reactive bands, and LAS-3000 mini (Fujifilm, Tokyo, Japan) chemiluminescence detection device was use to visualize the labels.

### Caspase activity assay

The Apo-ONE® Homogenous Caspase-3/7 assay (Promega, WI, USA) was used according to the manufacturer's instructions to assess caspase-3 and caspase-7 activation. In brief, cells were seeded at 5.0×10^3^ cells per well in 96-well plates. After miRNA transfection and/or GEM treatment, Apo-ONE® Homogeneous Caspase-3/7 reagent was mixed, and the cells were then incubated at room temperature for 18 hr in the dark. A fluorescence reader, TECAN Infinite F500 (Tecan, Männedorf, Switzerland), was used to measure caspase-3/7 activation via the fluorescence reagent in the reaction mixture.

### Statistical Analysis

StatView version 5.0 for Windows (Stata Corp., College Station, Tex) was used to perform statistical analysis. The results were represented as means ± standard deviation. The Student t test was used to compare means from different groups; p values <0.05 were regarded as statistically significant.

## Results

### Sensitivity of each CCA cell line to Gem treatment

**Figure 1:**
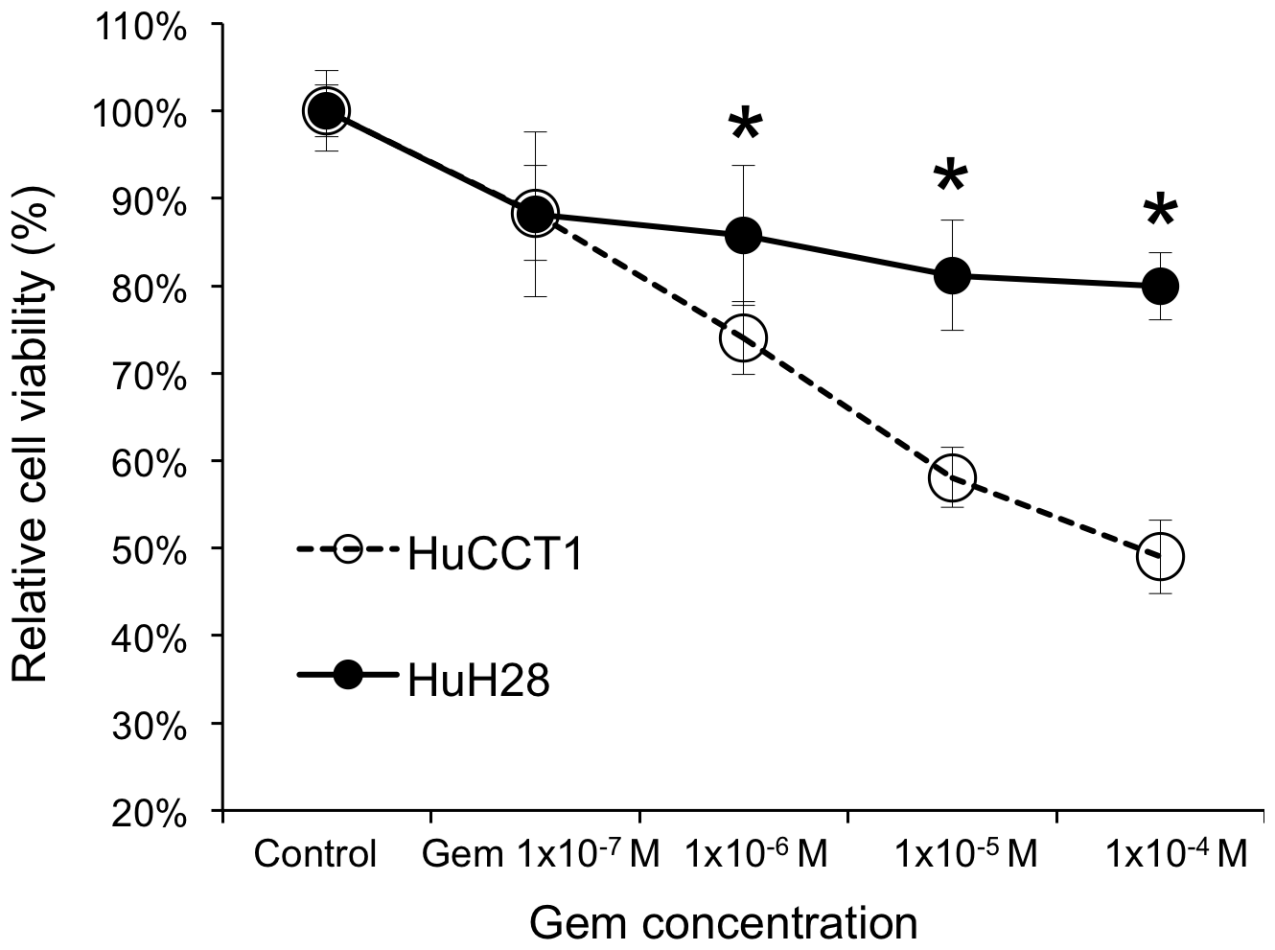
Sensitivity of two CCA cell lines, HuCCT1 and HuH28, to gemcitabine (Gem). HuCCT cells were significantly more sensitive than HuH28 cells to gemcitabine. *: p<0.05

First, we compared HuH28 and HuCCT1 with regard to sensitivity to Gem; we used CCK-8 assay to measure cell viability following Gem treatment. At concentrations of Gem near those used for clinical treatments (0.2−0.6×10^−4^ M), HuH28 cells were significantly less sensitive to Gem than were HuCCT1 cells [15] (Figure 1). After incubation in 1×10^−4^ M Gem for 72 hr, the relative cell viabilities of HuH28 and HuCCT1 were significantly different (p<0.0001) at 80 ± 4 % and 51 ± 4 % viability relative to control cells, respectively. The half maximal inhibitory concentration (IC50) of Gem in HuCCT1 and HuH28 cells were (8.7 ± 1.2)×10^−5^ M and over 1.0×10^−3^ M, respectively.

### miRNA expression profiles of HuCCT1 and HuCCT1cells

**Figure 2:**
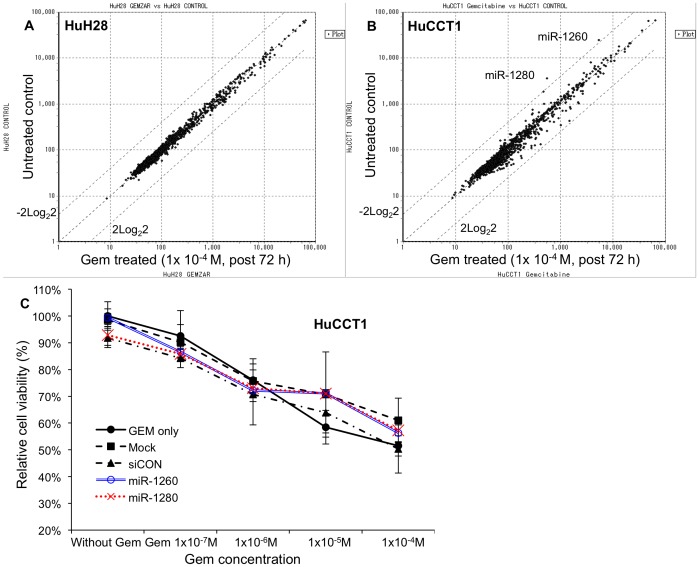
Gem treatment slightly affected miRNA expression profiles of CCA cell lines. (A) Scatter plot of miRNA expression log2 ratios between untreated and gemcitabine (Gem)-treated HuH28 cells. No miRNAs were differentially expressed because of the Gem treatment. (B) Scatter plot of miRNA expression log2 ratio between untreated and Gem-treated HuCCT1 cells. miR-1260 and miR-1280 were downregulated in Gem-treated HuCCT1 cells. (C) Ectopic overexpression of miR-1260 or miR-1280 by transfection of miRNA mimics did not affect the sensitivity of HuCCT1 cells to Gem. Relative cell viabilities were assessed 72 hr after Gem treatment. Final concentration of each miRNA mimic was 10 nM. Mock: receiving only transfection reagent. siCON: control treated with a non-silencing miRNA mimic.

To understand the roles of miRNAs in Gem resistance, we compared the miRNA expression profile of Gem-treated cells with that of untreated cells for each CCA cell line; in each case, the final Gem concentration was 1×10^−4^ M, and the incubation time was 72 hr. In HuH28 cells, no miRNA exhibited a changed in expression ratio greater than ± 2 log_2_ 2 in response to Gem treatment (Figure 2A). In HuCCT1 cells, the expression levels of two miRNAs, miR-1260 and miR-1280, were lower in Gem-treated cells than in untreated cells (normalized log_2_ ratio: −2.24 and −2.66, respectively. Figure 2B). Therefore, we used miRNA mimic transfection to examine the influence of miR-1260 and of miR-1280 on Gem sensitivity (Figure 2C). However, when miR-1260 or miR-1280 was overexpressed by transfection of the respective mimic miRNA, the relative cell viability of Gem-treated mimic-transfected cells did not differ from that of Gem-treated mock-transfected cells or of Gem-treated cells transfected with a scrambled control siRNA.

**Figure 3:**
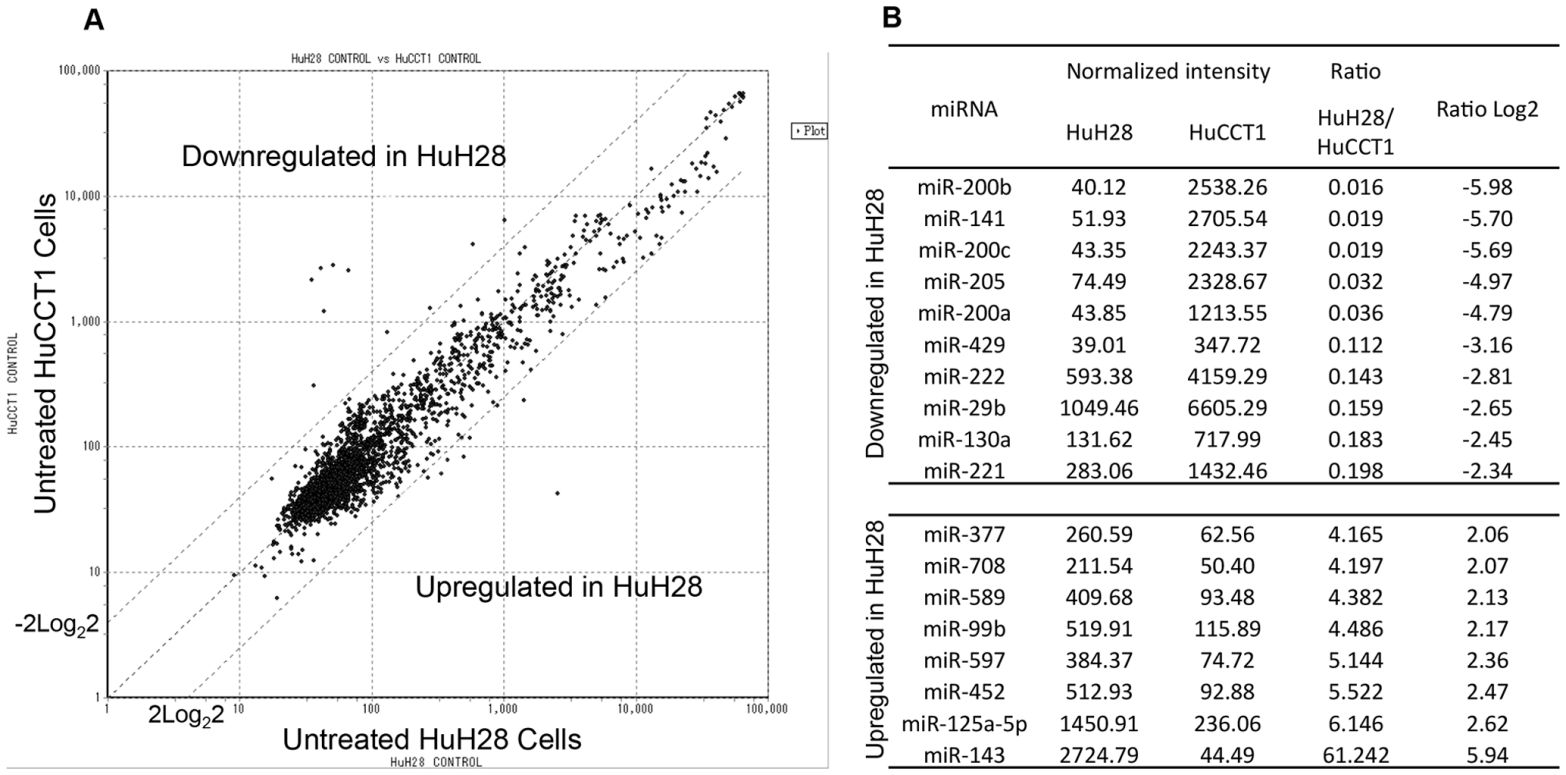
Eighteen miRNAs were differentially expressed between untreated HuH28 and untreated HuCCT1 cells. (A) Scatter plot of miRNA expression log2 ratios between untreated HuH28 and HuCCT1 cells. The threshold defining differential expression was a ratio smaller than -2log_2_2 or larger than 2log_2_2. (B) Normalized expression intensities and ratio values of the 18 miRNAs were reported in the table.

**Figure 4:**
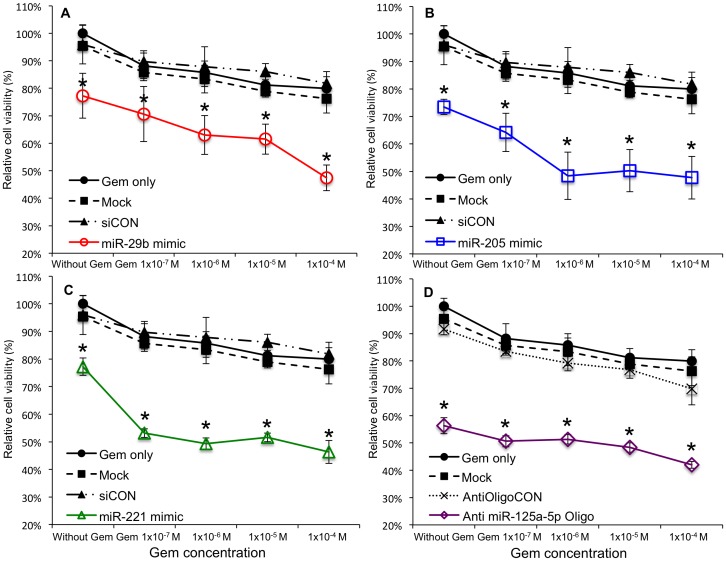
Modification of four of the candidate miRNA expression restored Gem sensitivity to HuH28 cells. Ectopic overexpression of miR-29b (A), miR-205 (B), or miR-221 (C) via transfection of a corresponding miRNA mimic and downregulation of miR-125a-5p (D) via transfection of an anti miRNA oligonucleotide made HuH28 cells more sensitive to Gem. Relative cell viabilities were assessed 72 hr after Gem treatment. The final concentration of each miRNA mimics was 10 nM; that of the anti miRNA oligonucleotide was 40 nM. Mock: receiving only transfection reagent. siCON: control treated with a non-silencing miRNA mimic. AntiOligoCON: control treated with a non-silencing control oligonucleotide. The asterisk denotes p<0.05 as compared to non-treated control, mock and siCON or control AntiOligoCON.

Next we compared the HuH28 and HuCCT1 cell lines with regard to their innate miRNA expression profiles. Expression of 18 miRNAs each differed between the two cell lines by a factor larger than ± 2 log_2_ 2 (Figure 3). Because HuCCT1 cells were more sensitive to Gem than were HuH28 cells, HuCCT1 cells were used as the standard in this comparison; expression of 10 miRNAs (miR-29b, 130a, 141, 200a, 200b, 200c, 205, 221, 222 and 429) was downregulated in HuH28 cells, while expression of eight others (miR-99b, 125a-5p, 143, 377, 452, 589, 597, and 708) was upregulated. To determine the role of each of the 18 candidate miRNAs in Gem resistance, we modified the expression level of each miRNA in HuH28 cells. The expression of each of the 10 downregulated miRNAs was enhanced in HuH28 cells by transfection of synthesized miRNA mimics; expression of each of the eight upregulated miRNAs was suppressed in HuH28 cells introduction of targeted anti-miRNA oligonucleotides. Transfection of a mimic of miR-29b, miR-205, or miR-221 or inhibition of miR-125a-5p via a complementary oligonucleotide significantly restored Gem sensitivity to HuH28 cells near clinical therapeutic concentration, 1×10^−4^ M (Figure 4). The relative cell viabilities were 47 ± 5 %, 48 ± 8%, 46 ± 4 % and 42 ± 1 % of untreated control, respectively. The p values between miR-29b, miR-205 and miR-221 mimic transfection versus non-silencing miRNA mimic (relative cell viability was 82 ± 4 % at 1×10^−4^ M Gem) and anti-miR-125a-5p oligonucleotide transfection versus negative control oligonucleotide (relative cell viability at 1×10^−4^ M Gem was 70 ± 6 %) were smaller than 0.001. These four miRNA modifications also significantly decreased cell viability over a broad range of Gem concentrations to a 0.001-fold lower of clinical therapeutic concentration (Figure 4A, B, C, D).

### Predicting the gene targets of miRNAs that affected Gem resistance

**Figure 5:**
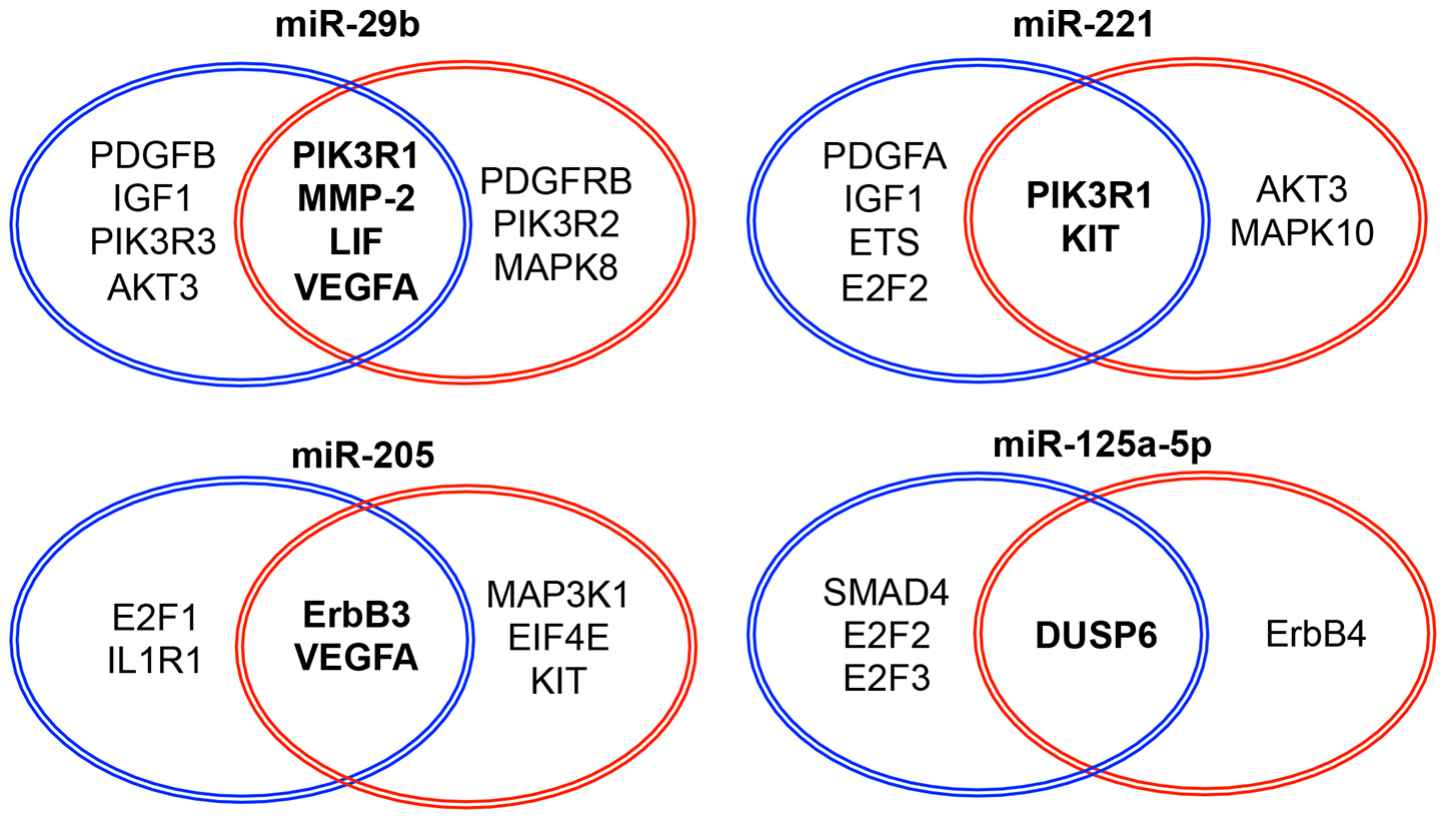
Web-driven programs predicted putative chemo-sensitivity-related target genes of candidate miRNAs. Genes that were among the ontology-filtered results from TargetScanHuman (left circle) and those from microT (right circle) were designated putative target genes of candidate miRNAs. Ontology filtering was performed with web-driven gene ontology software DIANA mirPath.

**Figure 6:**
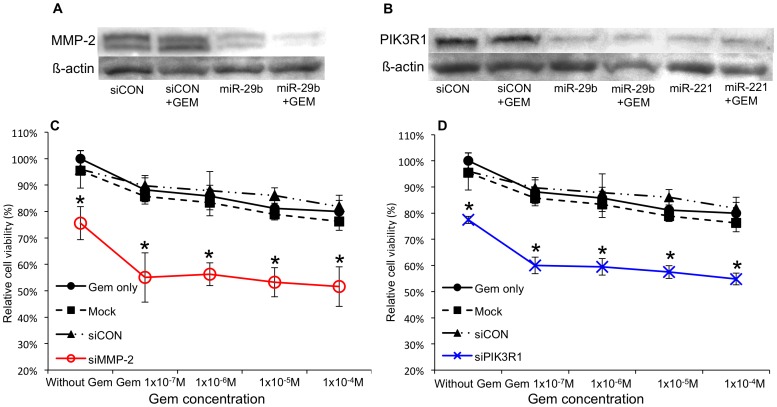
Two of software predicted miRNA target genes actually suppressed by corresponding miRNA. (A) MMP-2 and (B) PIK3R1 were significantly suppressed by transfection of the respective miRNA mimics. Selective downregulation of MMP-2 (C) or PIK3R1 (D) by transfection of each corresponding siRNA conferred Gem sensitivity to HuH28 cells. The analysis of Western blot and relative cell viability were performed 72 hours after Gem treatment. Final concentration of siRNAs and miRNA mimics were 10 nM. Mock: receiving only transfection reagent. siCON: control treated with a non-silencing miRNA mimic. The asterisk denotes p<0.05 as compared to non-treated control, mock and siCON.

A single miRNA could potentially regulate the expression of hundreds of target genes simultaneously. To examine in more detail the function of miRNAs in the sensitivity of CCA to Gem treatment, we used computational analyses to search for the gene targets of these four miRNAs that affected the sensitivity of HuH28 cells Gem (Figure 5). Web-driven programs were used to identify putative genes target of these four miRNAs and to identify those putative targets that might be related to chemosensitivity. The genes that were identified as potential targets by both programs, TargetScanHuman 5.1 and microT 4.0, and were among the ontology-filtered results of both programs were defined as putative target genes that may be related to chemosensitivity. Based on these analyses, we predicted that six genes— erythroblastic leukemia viral oncogene homolog 3 (ERBB3), KIT, Leukemia inhibitory factor (LIF), matrix metalloproteinase 2 (MMP-2), phosphoinositide-3-kinase regulatory subunit 1 (PIK3R1) and vascular endothelial growth factor A (VEGFA) — were putative oncogenic targets of miR-29b, miR-205, and/or miR-221. Dual specificity phosphatase 6 (DUSP6) was predicted to be the putative anti-oncogene target of miR-125a-5p (Figure 5). We analyzed the expression of each of the six putative target genes after having modified the expression of each of the respective microRNAs; again, a miRNA mimic for miR-29b, miR-205, or miR-221 was transfected into cells to mimic miRNA overexpression; separately, an anti-miR-125a-5p oligonucleotide was transfected into cells to inhibit miR-125a-5p activity. Only two potential targets, PIK3R1 and MMP-2, were significantly suppressed by altering the expression of a miRNA; specifically, overexpression of miR-29b or miR-221 suppressed PIK3R1, and overexpression of miR-29b suppressed MMP-2. (Figure 6A, B). Other 4 target expression levels were not affected by corresponding miRNA expression modifications (Figure S1). We then suppressed the expression of PIK3R1 or MMP-2 in HuH28 cells by transfection of a corresponding siRNA (Figure S2). Selective siRNA-mediated downregulation of PIK3R1 or MMP-2 conferred Gem sensitivity to HuH28 cells (Figure 6C, D). These results were the same as the results of overexpressing the respective miRNAs. In addition, we referred the baseline expression levels of PIK3R1 and MMP-2 between HuH28 and HuCCT1 cells by cancer cell line encyclopedia (CCLE; http://www.broadinstitute.org/ccle), the open access web database. In HuH28 cells, PIK3R1 and MMP-2 were more abundantly expressed than HuCCT1 cells. The normalized Log2 values were 0.92451 versus −0.42455 and 7.1731 versus −0.31517, respectively. We also used CCLE web software to check the mutations of the target genes. Both of HuH28 and HuCCT1 cells did not have any gene polymorphisms in PIK3R1 and MMP-2. Based on these findings, we reasoned that miR-29b and miR-221 restored Gem sensitivity to HuH28 cells, at least in part, by suppressing PIK3R1, MMP-2, or both; specifically, miR-29b could potentially suppress both genes, while miR-221 could suppress only PIK3R1.

### Caspase 3/7 activity

**Figure 7:**
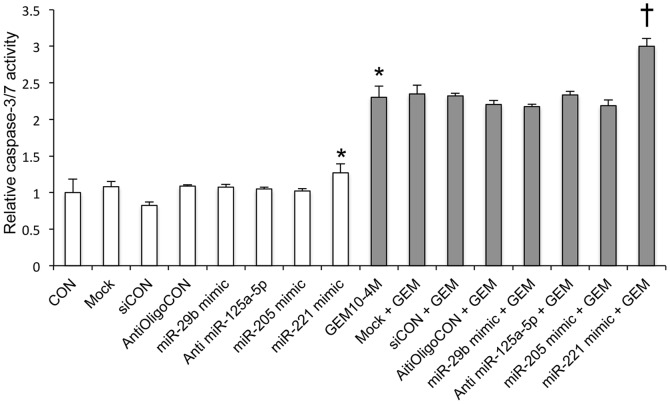
Caspase-3/7 activity assays were performed to assess apoptosis. The final concentrations of miRNA mimics and anti miRNA oligonucleotide were 10; the final concentration of Gem was 1×10^−4^ M. Mock: receiving only transfection reagent. siCON: control treated with a non-silencing miRNA mimic. *: p<0.05 versus non-treated control. †: p<0.05 versus Gem treated.

To assess the induction of apoptosis in Gem-treated HuH28 cells that had been subjected to changes in miRNA expression, caspase-3 and caspase-7 activity was assayed (Figure 7). Transfection of the miR-221 mimic resulted in an increase in caspase-3/7 activity (1.3 fold of control, p = 0.03) relative to the controls, but manipulation of any of three other miRNAs did not result in any differences in caspase activity. Gem treatment (1×10^−4^ M, 72 hours) resulted in a 2.3-fold increase in caspase-3/7 activity over that in controls (p<0.001 versus control), and transfection of the miR-221 mimic transfection and Gem treatment in combination caused caspase-3/7 activity to increase 3.0-fold over that in controls (p<0.001 versus Gem-treated cells).

## Discussion

By comparing the miRNA expression profiles of two 2 CCA cell lines, we identified a set of four miRNAs that affected the Gem sensitivity of the innately Gem-resistant HuH28 CCA cells.

We found that miR-29b was downregulated in the more GEM-resistant CCA cell line, HuH28, and that ectopic overexpression of miR-29b caused by transfection with a miRNA mimic conferred GEM sensitivity to the HuH28 cells. MicroRNA-29b is one of the representative anti-onco-miRNAs in many kind of cancers [16]–[18]. Here, we identified two miR-29b target genes, PIK3R1 and MMP-2, that are, at least partly, responsible for the resistance of CCA Gem treatment. PIK3R1 encodes phosphoinositide 3-kinase (PI3K) regulatory subunit designated p85 alpha; p85 alpha is regarded as integrator of multiple signaling pathways that together promote cell proliferation, cell survival, and carcinogenesis [19]. Inhibition of PIK3R1 promotes apoptosis by reducing PI3K-dependent signaling [19]. Stronach et al. have shown that PIK3R1 knockdown restored the sensitivity of ovarian cancer to platinum treatment [20]. MMP-2 is a member of the family of zinc-dependent endopeptidases; these enzymes share specific structural components [21]. MMP-2 is known to promote tumor invasion, remote metastasis, and angiogenesis by degrading components of the extracellular matrix, mainly type IV collagen [21]. Fang et al. revealed that miR-29b suppresses tumor cell invasion and metastasis by downregulating MMP-2 expression [22]. Moreover, MMP-2 promotes cell survival and proliferation by inhibiting the binding of death ligands to the surfaces of tumor cells [23], [24]. Our findings on MMP-2 seemed to be explained by direct anti-proliferation effects on HuH28 cells. Similar to our results, some previous results also indicate that miR-29b suppresses growth of a human uterine carcinoma line (HeLa cells) and of prostate cancer cells by downregulating p85 alpha and MMP-2, respectively [18], [25].

Our findings indicated that oligonucleotide-mediated selective suppression of miR-125a-5p strongly reduced CCA cell viability. However, unlike the effects of other miRNAs related to Gem sensitivity, the decreasing rate of cell viability by Gem treatment did not change comparing with Gem treatment only group (Figure 4D). In the strictest sense, miR-125a-5p should be considered an onco-miRNA in HuH28 cells, but miR-125a-5p apparently did not affect the Gem sensitivity of these cells within the range of Gem concentrations used for clinical treatments. Reportedly, miR-125a-5p is an anti-onco miRNA in HCC and gastric cancer [26], [27]. However, miR-125a-5p expression is significantly upregulated in lung squamous cell carcinoma relative to that in normal lung tissue [28] and miR-125a-5p expression was associated with enhanced the pathological stage and lymph node metastasis in non-small cell lung cancer [29]. Our computer-based analysis identified that the target of miR-125a-5p was DUSP6, which is an anti-oncogene; however, DUSP6 expression was not enhanced by transfection of the anti-miR-125a-5p oligonucleotide. The genes encoding the precursors to miR-125a-5p, miR-99b and let7e are located in a conserved gene cluster on Chromosome 19 in humans. In our study, miR-99b was also downregulated in HuH28 cells relative to its expression in HuCCT1 cells by a factor of more than 2log_2_2, but selective suppression of miR-99b did not significantly change the relative cell number ratio when anti-oligonucleotide-treated cells were compared with control oligonucleotide-treated cells; 72 hr after 1×10^−4^ M Gem treatment, relative cell viabilities were 69 ± 3 % and 70 ± 6 %, respectively (p value  = 0.37, Figure S3).

Our results clearly indicated that excess miR-205 could conferred Gem sensitivity to innately Gem-resistant CCA cells. Reportedly, miR-205 is both an anti onco-miRNA and an onco-miRNA [30]. As an anti onco-miRNA, miR-205 targets and suppresses zinc finger E-box binding homeobox 1/2 (ZEB1, 2), E2F transcriptional factor 1 (E2F1), ErbB3, and VEGFA [31]–[34]. Our computer-based search for miR-205 targets also indicated that ErbB3 and VEGFA were cancer-related target genes of miR-205 in HuH28 cells. However, ectopic overexpression of the miR-205 mimic did not change expression levels of ErbB3 or VEGFA. Some other target genes, which we did not identify, may play key roles in Gem sensitivity of CCA cells. We discuss the limitations of our study in a later paragraph.

Our result indicated that miR-221 was downregulated in Gem-resistant HuH28 cells, and that it acted as a potent enhancer of Gem sensitivity, at least partly, by downregulating PIK3R1 expression. miR-221 is one of the most abundant miRNAs in non-malignant biliary epithelial cells; in libraries made from these cells, miR-221 clones represents approximately 10% of all miRNA clones [35]. Karakatsanis et al. showed that miR-221 was down regulated in resected CCA tissues [36]. Meanwhile, miR-221 expression is relatively low in many tissues from other organs, and miR-221 is considered an onco-miRNA in various human cancers—including HCC, pancreas cancer, gastric cancer [37]–[39]. In malignant cells, overexpressed miR-221 increased cell proliferation and resistance to anti-tumor treatments by downregulating tumor suppressor genes such as cyclin-dependent kinase inhibitors p27, p57 and phosphatase and tensin homolog deleted from chromosome 10 (PTEN) [37], [40]. Frequently, a single miRNA may have very different roles in cancer progression, and these roles often depend on the cancer and its organ and/or tissue of origin. One miRNA can have numerous target genes, virtually hundreds to thousands. Moreover, a single mRNA can have multiple miRNA binding sites [41]. Because of the complex cross-talk within regulatory networks, the function of intracellular miRNAs depend greatly on the tissue-specific miRNA expression profiles [42]. The role of miR-221 in tumor proliferation and survival may have been attenuated by the cholangiocyte-specific pattern of miRNA expression.

A few published studies have examined the influence of miRNA on Gem sensitivity in CCA cells. Meng et al. reported that the expression levels of miR-21 and miR-200b correlate with Gem resistance in a CCA cell line (Mz-ChA-1) derived from gallbladder carcinoma [43]. miR-21 is one of the representative oncogenic miRNAs that target tumor suppressor genes such as programmed cell death 4 and phosphatase and tensin homolog [44], [45]. Many studies have shown that miR-21 expression is strictly restricted in non-malignant cells and is frequently overexpressed in cancer cells—including those from hepatocellular carcinoma, breast cancer, lung cancer, esophageal cancer, gastric cancer, colon cancer, or glioblastoma [46]. And the expression levels of miR-21 correlate with cancer clinical stage, chemotherapy resistance, and poor prognosis [46], [47]. Several studies of CCA show that miR-21 is overexpressed in tumor cell and that the miR-21 expression level is related to CCA cell proliferation [48], [49]. Our findings indicated that miR-21 was highly expressed in both the Gem-sensitive HuCCT1 and the Gem-resistant HuH28 lines (9.6-fold and 19.8-fold greater than the mean of all miRNA intensities, respectively). However, these elevations in expression did not fulfill our criterion (Log ratio was 1.04). Furthermore, non-malignant cholangiocytes and normal vascular endothelial cells reportedly express exceptionally high levels of miR-21 [36], [50]. Our results regarding miR-21 expression were not contradictory to those from former reports, but did not show clear relationship to Gem sensitivity of CCA cells possibly because of cholangiocyte-specific miRNA expression profile. In contrast to miR-21, miR-200b was markedly downregulated in the more Gem-resistant HuH28 cells (Figure 2). And the ectopic overexpression of miR-200b did not affect the Gem resistance of HuH28 (Figure S4). The relative cell viability of miR-200b mimic transfection and mock transfection were 75 ± 6 % and 76 ± 5 %, respectively (72 hours after 1×10^−4^ M Gem treatment, p = 0.89). Furthermore, all expression levels of miR-200 family; miR-200a, miR-200c, miR-141, and miR-429 were also downregulated in HuH28 cells (Figure 2). However, modification of their expression by transfection of a corresponding miRNA mimic did not influence Gem sensitivity of these cells with a 72 hr treatment in 1×10^−4^ M Gem (82 ± 2 %, 69 ± 4 %, 70 ± 3 % and 75 ± 3 %. p values versus mock are 0.07, 0.06, 0.08 and 0.75, respectively, Figure S4). The reason for this discrepancy is not clear. It is possible that the difference in miRNA expression profile between intrahepatic CCA and gall bladder cancer may affect the functional of these miRNA in these cancers.

We newly identified miRNAs that affected Gem sensitivity in CCA cells. However, our methods have some theoretical limitations. In comparing the miRNA expression profiles of HuH28 and HuCCT1, we adjusted the threshold ratio of miRNA expression to more than ± 2log_2_2 to select candidate miRNAs. By this criterion, we should have selected nearly the top 1 % of all measured human miRNAs (18 candidate miRNAs/1896 scanned miRNAs). However, this threshold might have been too high because many differences that fell below that threshold may have been real and important differences. Moreover, when predicting the gene targets of the candidate miRNAs, we adopted an intersectional method using two different software programs. Each program has specific algorithms that incorporate several factors such as complementarity or binding energy of miRNA to mRNAs [8], [51]. This method is commonly used to identify a reliable set of putative target genes; however, it involves the risk of missing some real target genes. Ontology selection was useful for identifying putative target genes that might be relevant to the cell functions we wanted to discuss. However, ontology selection can only identify genes whose functions have been identified.

In conclusion, our analysis of miRNA expression profiles in CCA cells revealed that miR-29b, miR-205, and miR-221 expression levels were related to the Gem resistance of HuH28 cells, and that ectopic overexpression of any one of these miRNAs could restore Gem sensitivity to these cells. miR-125a-5p was regarded as a representative onco-miRNA in the CCA cells, and ectopic selective downregulation of miR-125a-5p repressed CCA cell proliferation. We also used web-driven software to identify potential gene targets of these key miRNAs and found that two of virtually predicted miRNA target genes, PIK3R1 and MMP-2, were promising anti-tumor targets in CCA cell signal pathways. Our results indicated that expression levels of miR-29b, miR-125a-5p, miR-205, and miR-221 may be useful as diagnostic markers of sensitivity to Gem treatment, and that PIK3R1 and MMP-2 could become molecular targets of anti-tumor therapies for patients with CCA. Further studies using other intrahepatic CCA cells and in vivo are required in order investigating the association between these 4 miRNAs and the chemo-sensitivity of intrahepatic CCA closely.

## Supporting Information

Figure S1 **Some of predicted miRNA target gene expression levels were not affected by corresponding miRNA modifications.** Western blot analysis was performed to assess protein expression levels from the genes designated a putative miRNA targets. The expression levels of LIF, ERBB3, VEGFA and DUSP6 in HuH28 were not changed by transfection of the respective miRNA mimic or anti miRNA oligonucleotide. KIT did not express in HuH28 cells. siCON: control treated with a non-silencing miRNA mimic. Final concentration of miRNA mimics and siCON were 10 nM and anti miRNA oligonucleotide was 40 nM. The final concentration of Gem was 1×10^−4^ M. The analysis was performed at 72 hours after Gem treatment.
(TIF)

Figure S2 **Down-regulation of PIK3R1 and MMP-2 expression levels by corresponding siRNAs.** The expression of PIK3R1 and MMP-2 in HuH28 cells were suppressed by transfection of their corresponding siRNAs. siCON: control treated with a non-silencing miRNA mimic. The final concentration of Gem was 1×10^−4^ M. Final concentration of miRNA mimics and siCON were 10 nM. The analysis was performed at 72 hours after Gem treatment.
(TIF)

Figure S3 **Seven miRNAs which were upregulated in HuH28 did not relate to GEM sensitivity.** Relative cell viabilities were assessed 72 hr after Gem treatment. The final concentration of each anti miRNA oligonucleotide was 40 nM. Mock: receiving only transfection reagent. siCON: control treated with a non-silencing miRNA mimic. AntiOligoCON: control treated with a non-silencing control oligonucleotide.
(TIF)

Figure S4 **Seven miRNAs which were downregulated in HuH28 did not relate to GEM sensitivity.** Relative cell viabilities were assessed 72 hr after Gem treatment. The final concentration of each miRNA was 10 nM. Mock: receiving only transfection reagent. siCON: control treated with a non-silencing miRNA mimic.
(TIF)
