# Supplementary figures and images for: miR-29b, miR-205 and miR-221 Enhance Chemosensitivity to Gemcitabine in HuH28 Human Cholangiocarcinoma Cells
Source: PLoS One. 2013 Oct 17;8(10):e77623. doi: 10.1371/journal.pone.0077623 (PMC3798426; doi:10.1371/journal.pone.0077623)

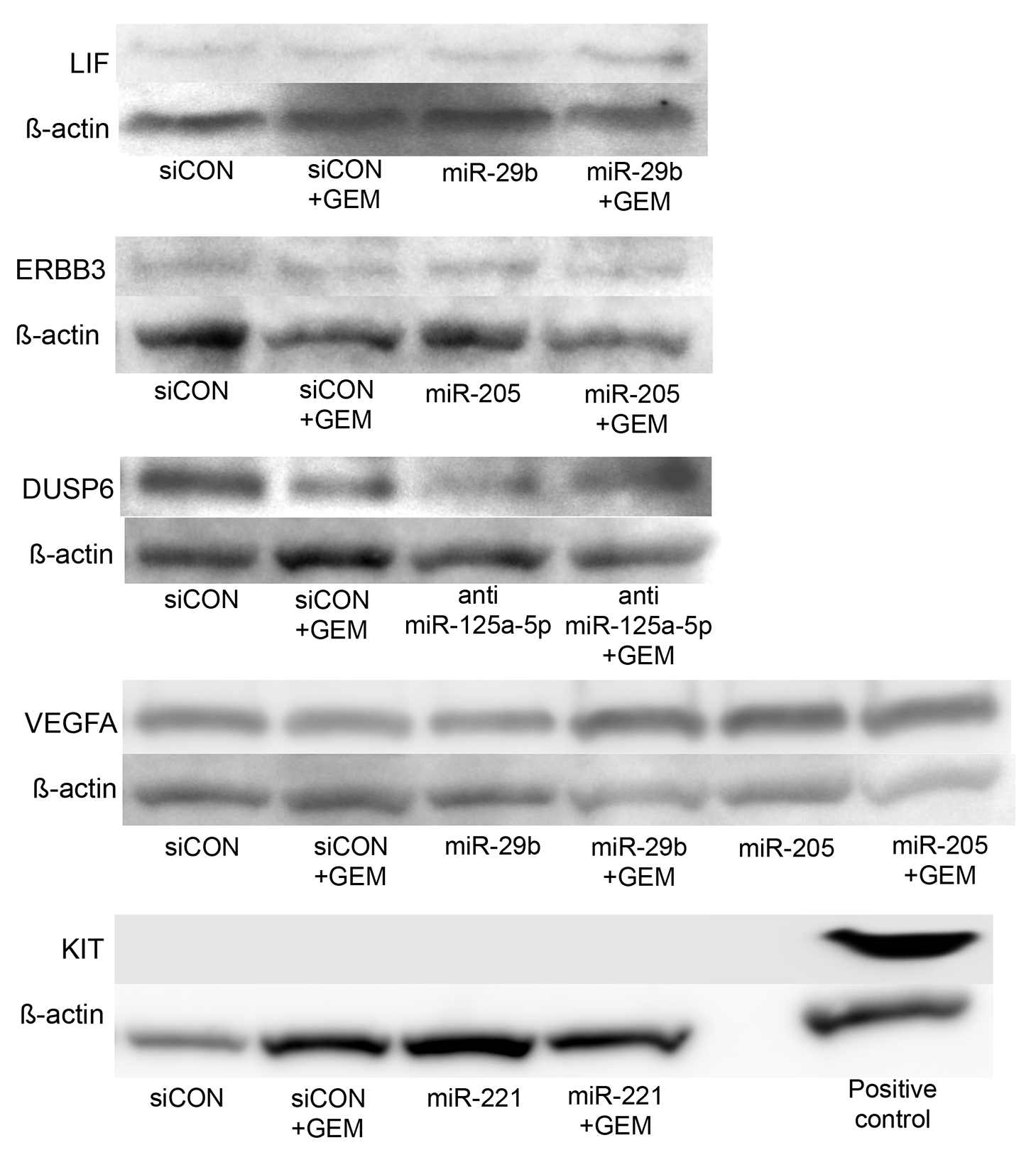

Supplement: Figure S1 — Some of predicted miRNA target gene expression levels were not affected by corresponding miRNA modifications. Western blot analysis was performed to assess protein expression levels from the genes designated a putative miRNA targets. The expression levels of LIF, ERBB3, VEGFA and DUSP6 in HuH28 were not changed by transfection of the respective miRNA mimic or anti miRNA oligonucleotide. KIT did not express in HuH28 cells. siCON: control treated with a non-silencing miRNA mimic. Final concentration of miRNA mimics and siCON were 10 nM and anti miRNA oligonucleotide was 40 nM. The final concentration of Gem was 1×10^−4^ M. The analysis was performed at 72 hours after Gem treatment. (TIF) [file pone.0077623.s001.tif]

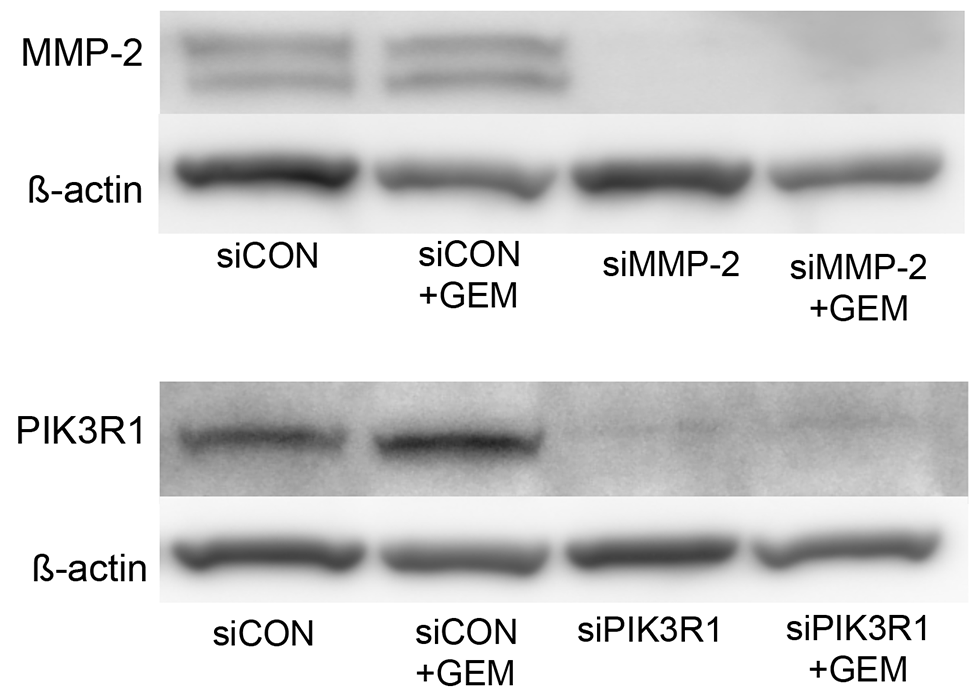

Supplement: Figure S2 — Down-regulation of PIK3R1 and MMP-2 expression levels by corresponding siRNAs. The expression of PIK3R1 and MMP-2 in HuH28 cells were suppressed by transfection of their corresponding siRNAs. siCON: control treated with a non-silencing miRNA mimic. The final concentration of Gem was 1×10^−4^ M. Final concentration of miRNA mimics and siCON were 10 nM. The analysis was performed at 72 hours after Gem treatment. (TIF) [file pone.0077623.s002.tif]

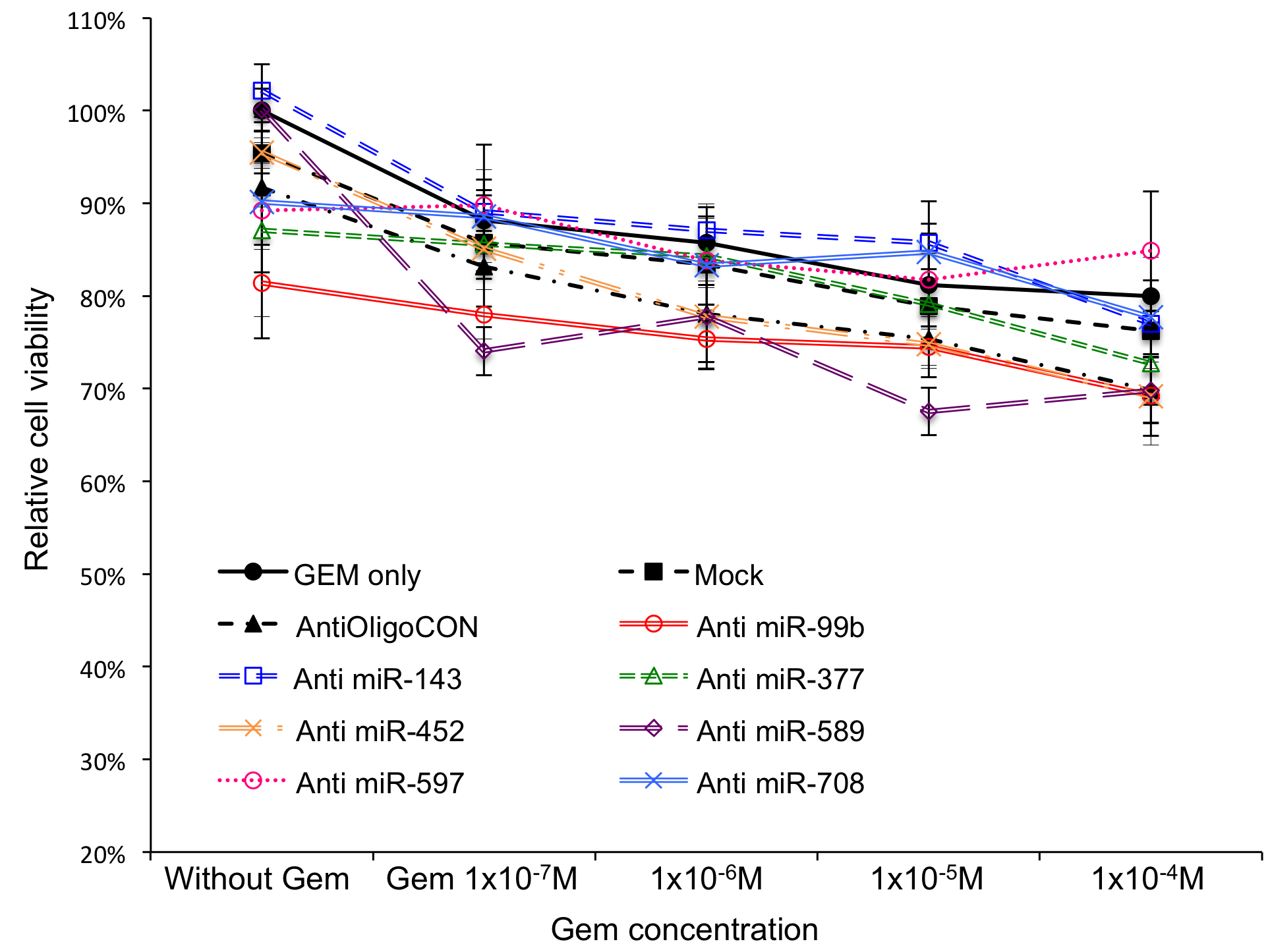

Supplement: Figure S3 — Seven miRNAs which were upregulated in HuH28 did not relate to GEM sensitivity. Relative cell viabilities were assessed 72 hr after Gem treatment. The final concentration of each anti miRNA oligonucleotide was 40 nM. Mock: receiving only transfection reagent. siCON: control treated with a non-silencing miRNA mimic. AntiOligoCON: control treated with a non-silencing control oligonucleotide. (TIF) [file pone.0077623.s003.tif]

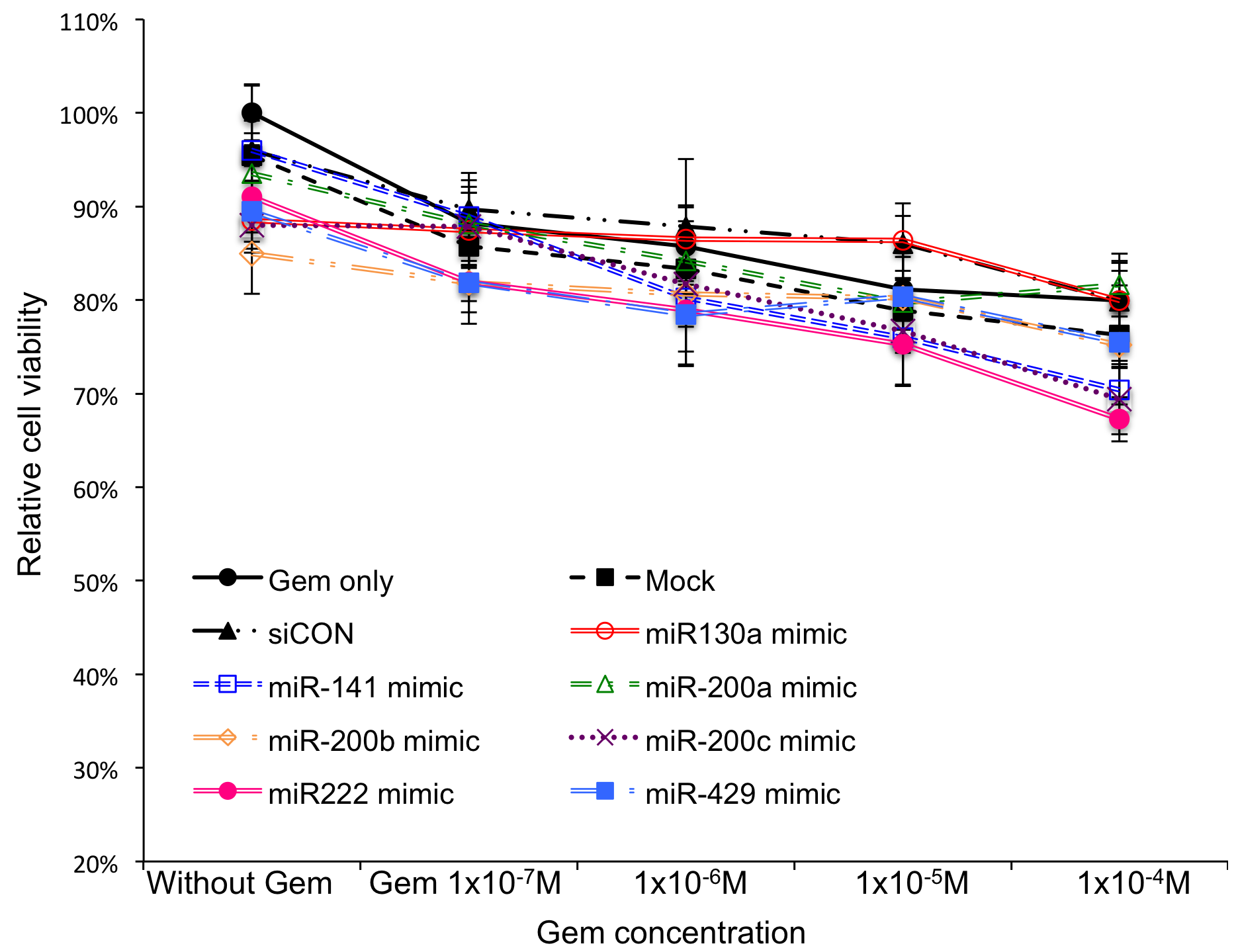

Supplement: Figure S4 — Seven miRNAs which were downregulated in HuH28 did not relate to GEM sensitivity. Relative cell viabilities were assessed 72 hr after Gem treatment. The final concentration of each miRNA was 10 nM. Mock: receiving only transfection reagent. siCON: control treated with a non-silencing miRNA mimic. (TIF) [file pone.0077623.s004.tif]
